# A Case of Acute Hæmorrhagic Pancreatitis

**Published:** 1906-01-20

**Authors:** 


### A CASE OF ACUTE HÆMORRHAGIC PANCREATITIS.

A very graphic record of this rare disease is contributed by Dr. Harold V. Munster.[1] The patient was a man of 54 years. With the exception of occasional attacks of abdominal pain during the preceding three years, he had enjoyed good health. On October 29, 1905, he had to send for medical advice in consequence of abdominal pain. The next day he was found much collapsed, with a pulse of 144, and complaining much of thirst. Vomiting was frequent, and the vomit often contained "black blood"; the bowels had acted under the influence of castor oil. The temperature was normal. Later, diarrhœa was troublesome, but after a few days the patient improved to such an extent that his recovery was confidently anticipated. Unfortunately, unfavourable symptoms soon appeared—bleeding from the rectum, retention of urine, and delirium. Coma shortly supervened, and death occurred on November 9—that is, 11 days from the initial seizure.

At the post-mortem examination nodules showing fat necrosis were distributed widely over the peritoneum. The pancreas was much enlarged, and was studded with hæmorrhages, though the gland tissue was by no means obliterated. The tendency to hæmorrhage, as shown by the hæmatemesis, and the discharge of blood from the bowel is a noteworthy feature of the case; also the state of the bowels, constipation at first, followed by diarrhœa. The relatively long duration of the case is also of interest.

[1] Lancet, Dec. 30, 1905.

